# EEG-Based Automatic Sleep Staging Using Ontology and Weighting Feature Analysis

**DOI:** 10.1155/2018/6534041

**Published:** 2018-09-04

**Authors:** Bingtao Zhang, Tao Lei, Hong Liu, Hanshu Cai

**Affiliations:** ^1^School of Information Science and Engineering, Lanzhou University, Lanzhou, China; ^2^School of Electronic and Information Engineering, Lanzhou Jiaotong University, Lanzhou, China; ^3^College of Electronical and Information Engineering, Shaanxi University of Science and Technology, Xi'an, China; ^4^School of Information Science and Engineering, Shandong Normal University, Jinan, China

## Abstract

Sleep staging is considered as an effective indicator for auxiliary diagnosis of sleep diseases and related psychiatric diseases, so it attracts a lot of attention from sleep researchers. Nevertheless, sleep staging based on visual inspection of tradition is subjective, time-consuming, and error-prone due to the large bulk of data which have to be processed. Therefore, automatic sleep staging is essential in order to solve these problems. In this article, an electroencephalogram- (EEG-) based scheme that is able to automatically classify sleep stages is proposed. Firstly, EEG data are preprocessed to remove artifacts, extract features, and normalization. Secondly, the normalized features and other context information are stored using an ontology-based model (OBM). Thirdly, an improved method of self-adaptive correlation analysis is designed to select the most effective EEG features. Based on these EEG features and weighting features analysis, the improved random forest (RF) is considered as the classifier to achieve the classification of sleep stages. To investigate the classification ability of the proposed method, several sets of experiments are designed and conducted to classify the sleep stages into two, three, four, and five states. The accuracy of five-state classification is 89.37%, which is improved compared to the accuracy using unimproved RF (84.37%) or previously reported classifiers. In addition, a set of controlled experiments is executed to verify the effect of the number of sleep segments (epochs) on the classification, and the results demonstrate that the proposed scheme is less affected by the sleep segments.

## 1. Introduction

Sleep [1] is one basic need for the human, both in terms of physical and mental health. However, nowadays many countries around the world have been into aging society, there have been significant and growing incidences of sleep-related conditions such as obstructive sleep apnea (OSA), insomnia [2], and narcolepsy [3]. Generally, these sleep-related conditions may cause depression [4, 5], irritation, anxiety, or even death, so on and so forth, which seriously affect the quality of life (QoL) for those suffering from such conditions [6].

Accurate and effective identification of sleep staging is an important step in assisting the diagnosis of sleep-related disorders. Traditionally, all night polysomnographic (PSG) recordings are visually scored by well-trained experts based on Rechtschaffen and Kales's (R&K) recommendations [7] or a new guideline developed by the American Academy of Sleep Medicine (AASM) [8]. PSG is a comprehensive record of concurrent physiological signal during sleep, and this record principally includes EEG, electrooculogram (EOG), chin electromyogram (EMG), electrocardiogram (ECG), oxygen saturation (SpO_2_), respiration (Resp), and rectal body temperature. The most important signal for sleep staging is EEG from PSG because the clinically acceptable sleep staging is mainly determined by EEG [9, 10]. Thus this study extracts features derived from EEG data. The typical recording period of EEG segment is divided into 20 or 30 seconds prior to being classified as different sleep stages. We use a fixed 30s segment in this study. These different sleep stages and their relationship with EEG signals are briefly described as follows [1, 6, 7, 11, 12]:

1. *Wakefulness (WA)*: it is an awakening state of subject before falling asleep. In this stage, the EEG signal rapidly changes, and alpha waves (8–13 Hz) are most significant.
2. *Nonrapid eye movement (NREM) sleep stage 1 (NREM1)*: this first stage of sleep is also called the drowsiness state. During this stage, brain activity slows down and theta waves (4–8 Hz) are more prominent. Meanwhile, the eyes begin to roll slightly.
3. *NREM sleep stage 2 (NREM2)*: in this stage, the EEG amplitude becomes higher, sleep spindles (11–15 Hz), and K-complexes appear. Meanwhile, the eyes stop moving.
4. *NREM sleep stage 3 (NREM3)*: this sleep stage is also called the deep sleep. In this stage, 20% to 50% of EEG signals are delta waves (2–4 Hz) and the rest are theta waves. The people may experience sleepwalking, night terrors, and bedwetting during this period.
5. *NREM sleep stage 4 (NREM4)*: this stage is a continuation of deep sleep. And it also called the slow wave sleep. In this stage, more than 50% of EEG frequency varies between 0.5 Hz and 2 Hz (delta wave).
6. *Rapid eye movement (REM)*: the eyes remain closed but move rapidly. Beta waves are more predominant and frequency is greater than 13 Hz.

In addition, there are five different combination ways (CWs) for the above six sleep stages according to the R&K standard (Table 1) [10].

Sleep staging on the basis of visual EEG interpretation is problematic for many reasons [9, 13]. The accuracy of sleep staging depends on subjective experience of experts. Furthermore, research shows that the classification consistency among experts is less than 85%. Meanwhile, the analysis of the huge bulks of data from patients makes sleep staging by human experts become onerous and subject to misclassification due to fatigue. In clinical settings, there is a certain lag in sleep staging based on visual inspection, due to its time-consuming process. To solve these problems, it is obvious that there is a great need of an automatic sleep-staging scheme.

With the continuous development of new technology, the ability of EEG to automatically identify sleep staging is increased because a large number of features extracted from EEG data. A huge number of features enhance classification accuracy of sleep staging. However, to manage a large number of unstructured features is a very complex task for data management professionals. Hence, we believe that as an essential condition for an automatic sleep-staging scheme, automatic management and representation of EEG features is necessary. In this situation, ontology techniques exhibit several benefits in terms of information representation, and it has the major advantages as follows [14]: (1) it can realize the effectiveness, specification, and conciseness of information representation; (2) it can manage a large number of features in a hierarchical manner.

The remainder of this paper is organized as follows: related research is considered in Section 2 with data description introduced in Section 3.  Section 4 describes the proposed EEG-based automatic sleep-staging scheme. In Section 5, we set out experimentation with the results and a discussion. Finally, the conclusion and future work are presented in Section 6.

## 2. Related Research

Automatic sleep staging has become a hot issue in sleep research field; some studies emphasize the importance of selecting a suitable classifier to improve classification accuracy. Such research involves support vector machines [6, 15], neural networks [12, 16, 17], random forest [18], decision trees [19], Hidden Markov Models [20], and visibility graphs [13, 21, 22]. Sharma et al. [6] have developed a single-channel EEG-based sleep stages classification system using a novel three-band time-frequency localized (TBTFL) wavelet filter bank (FB). The system achieved classification accuracies from 91.5% to 98.3% for five CWs. In [12], the power spectral density (PSD) features extracted from multichannel neural data were fed into a multilayer feedforward network classifier for five-state sleep classification, and the classification accuracy of 90.1% was obtained. Hsu et al. [17] present a recurrent neural classifier for automatically classifying sleep stages based on energy features from EEG signal. Zhu et al. [13] analyzed sleep stages based on visibility graphs. The representative graph features were extracted and then were forwarded to a classifier to distinguish different sleep stages. The datasets used in the research were from the Sleep-EDF database, and the classification accuracy of 87% was reported. Diykh et al. [21, 22] proposed a novel method of sleep stages classification based on complex networks, and its method is mainly composed of two steps: (1) Each EEG segment is partitioned into subsegments. The size of a subsegment is determined empirically. (2) Statistical features are extracted, sorted into different feature sets, and then forwarded to the structural graph similarity and the K-means (SGSKM) to classify EEG sleep stages.

To better introduce the research status of automatic sleep staging based on EEG, the following is an overview of some representative studies in recent years. Acharya et al. [1] gave a comprehensive and comparative analysis of 29 nonlinear dynamic measures for EEG-based sleep stages classification. An iterative filtering-based decomposition is presented for automatic classification of sleep stages using EEG signals [10]. In [11], a number of higher order spectra- (HOS-) based features were extracted from unique bispectrum and bicoherence during the different sleep stages, and then these features were fed to a Gaussian mixture model (GMM) classifier for automatic sleep staging. Liang et al. [9] proposed an automatic sleep-scoring method combining multiscale entropy (MSE) and autoregressive (AR) models. Berthomier et al. [23] proposed an approach which uses an iterative adaptive fuzzy logic system to iterative update sleep stage pattern definitions. The above three studies have achieved an average accuracy for five-state sleep stages which are 83.6%, 83.49%, and 71.2%. An intelligent system for sleep stages classification (ISSSC) was designed and implemented [24]. The system consists of four principal modules: signals preprocessing, machine learning, inference, and inference corrector module.

Though many research results of sleep stages based on EEG have been reported in the literature, these studies have differing advantages and limitations. None of them have been pervasively applicable, maybe it is because of a lack of effective mechanism for presentation and management of the large number of EEG features. In our opinion, two important problems should to be solved in order to get an acceptable scheme of automatic sleep staging: (1) the scheme should be able to express and manage related information of sleep staging (such as a large number of unstructured EEG features) using a few simple terms which are readable by humans and computers. Simultaneously, the related information of sleep staging can be processed by computers easier and faster; (2) the scheme should be able to provide a valid analysis and classification method for EEG signals and sleep staging.

OBM [25] as a tool has been applied in many fields of information representation. Such as affective computing [26–28], attack detection [29], agriculture [30], and intelligent transport [31]. In addition, OBM has also been applied in expert system; for example, Bau et al. [32] construct a clinical decision support system (CDSS) for undergoing surgery based on ontology and rules reasoning in the setting of hospitalized diabetic patients. Wang et al. [33] proposes an assisting support system based on ontology, which is used to embody the relations among knowledge points. However, these studies rarely involve the use of ontology to describe and manage physiological features.

The purpose of this study is to improve the accuracy of sleep staging and fill the gap in the field of massive EEG feature representation. So based on inspiration of above research, this study proposes an automatic sleep-staging scheme based on EEG, which employs OBM to represent and organize related information of sleep staging and weighting feature analysis to improve the existing classification algorithm. Before ending this section, the main novelties of this paper are presented as follows:

1. An ontology-based model (OBM) is designed and applied to manage a high volume of sleep EEG features and context information.
2. An improved method of self-adaptive correlation analysis is designed to select the most effective EEG features.
3. Based on weighting features analysis, the improved RF is considered as the classifier to achieve the classification of sleep stages.
4. The effectiveness of the proposed method is validated by several sets of experiments.

## 3. Data Description

The raw physiological data are obtained from the Sleep-EDF database (EXPANDED) [34, 35], which contains 61 data recordings taken from 42 Caucasian subjects. The first 39 data records (SC∗PSG.edf files) are obtained from 20 healthy volunteers without any sleep-related disease. At the time of recordings, the demographic range was 25 to 34 years with the population consisting of 10 males and 10 females. The remaining 22 data records (ST∗PSG.edf files) are collected from 22 participants consisting of 7 males and 15 females aged from 18 to 79; all 22 subjects experienced mild difficulty in falling asleep.

The database includes dual-channel EEG from Fpz-Cz and Pz-Oz with a sampling rate of 100 Hz. Meanwhile, this database also contains other physiological signals, such as EOG, EMG, oronasal respiration, and so on. In this study, we only use two-channel EEG data (Fpz-Cz and Pz-Oz) to realize automatic sleep staging because of EEG is derived from the central nervous system, and it is most effective for the sleep stages classification.

To ensure the equality of 61 data records, 840 sleep segments (the length of each segment is 30 seconds) are randomly selected from each data record. So the total numbers of sleep segments are 51240. Specific database usage is shown in Table 2.

## 4. Methods

### 4.1. Our Work

**Figure 1:**
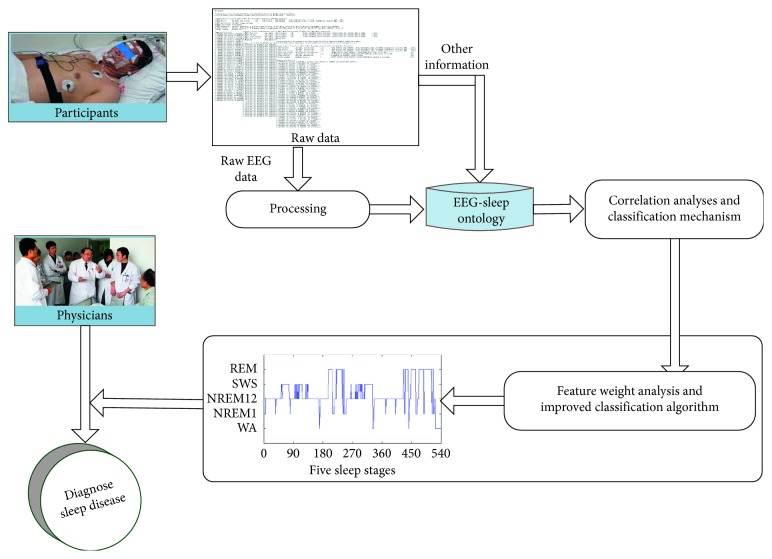
The structure diagram of our proposed scheme.

Figure 1 provides a structure diagram of our proposed scheme. The simplified execution process is shown as follows:

1. Raw physiological data are collected during sleep. The EEG in raw physiological data cannot be used directly for sleep staging, so first of all is EEG preprocessing. Meanwhile, other sleep-related information is directly mapped to “EEG-Sleep ontology.”
2. A large number of preprocessed EEG data are also mapped to “EEG-Sleep ontology.” It means that these EEG features together with sleep-related information are used to form the “EEG-Sleep ontology.”
3. By correlation analysis of the information stored in “EEG-Sleep ontology,” the subsets of EEG features that are most relevant to the sleep staging are obtained.
4. Next, the classification mechanism is introduced, and then an analysis strategy of the weighting feature is proposed to improve the classification algorithm.
5. The improved classification algorithm is used to infer different sleep stages. Finally, the results of sleep staging will be used to assist physicians in the diagnosis of sleep disorders.

### 4.2. Data Preprocessing

Usually, for the accurate analysis of data, it is necessary to preprocess the raw EEG data. Research [17] shows that the frequency range associated with sleep stages occur in 0.5 Hz to 30 Hz. Therefore, a 0.5 high-pass filter removed the low-frequency drift, and a 30 Hz low-pass filter removed the high frequency noise. Ocular artifacts appeared in the frequency ranges from 0 to 16, thus overlapping with other frequency bands, such as theta (4–8 Hz), alpha (8–13 Hz), and so on. So this study uses a FastICA [36, 37] to denoise overlapping frequency bands.

Traditionally, EEG features are extracted using the linear analysis method, which can be used to simulate a time series of signals with a specific mathematical formula in several nonoverlapping frequency bands. Recently, various nonlinear methods have also been widely used to extract EEG features. Because the EEG signals are derived from the central nervous system it is necessary to combine linear and nonlinear methods for EEG signal processing.

#### 4.2.1. Linear Method

In this study, 4 linear features of EEG signal are extracted from delta (0.5–2 Hz), sawtooth (2–4 Hz), theta (4–8 Hz), alpha (8–13 Hz), beta (13–30 Hz) bands: absolute power, relative power, center frequency, and max power. In addition, Hjorth parameters (Activity, Mobility, and Complexity), Skewness, and Kurtosis are also extracted from EEG signals in the full-frequency range (0.5–30 Hz) to dig the statistical properties and asymmetry of EEG. Among them, Activity is calculated as follows:

(1) $$\begin{array}{c} \text{Act}=\frac{1}{n-1}\overset{n}{\underset{i=1}{\sum }}{\left(x\left(i\right)-\mu \right)}^{2}. \end{array}$$

Mobility is calculated as follows:

(2) $$\begin{array}{c} \text{Mob}=\sqrt{\frac{\left(\left(1/\left(n-2\right)\right){\sum_{i=2}^{n}\left(x_{\text{d}}\left(i\right)-\mu_{\text{d}}\right)}^{2}\right)}{\left(1/\left(n-1\right)\right){\sum_{i=1}^{n}\left(x\left(i\right)-\mu \right)}^{2}}}. \end{array}$$

Complexity is calculated as follows:

(3) $$\begin{array}{c} \text{Cpx}=\sqrt{\frac{\left(\left(1/\left(n-3\right)\right){\sum_{i=3}^{n}\left(x_{\text{dd}}\left(i\right)-\mu_{\text{dd}}\right)}^{2}\right)}{\left(1/\left(n-1\right)\right){\sum_{i=1}^{n}\left(x\left(i\right)-\mu \right)}^{2}}}. \end{array}$$

Skewness is calculated as follows:

(4) $$\begin{array}{c} \text{Skew}=\frac{\left(1/n\right)\sum_{i=1}^{n}{\left(x\left(i\right)-\mu \right)}^{3}}{{\left(\sqrt{\mathrm{var}}\right)}^{3}}. \end{array}$$

Kurtosis is calculated as follows:

(5) $$\begin{array}{c} \text{Kurt}=\frac{\left(1/n\right)\sum_{i=1}^{n}{\left(x\left(i\right)-\mu \right)}^{4}}{{\mathrm{var}}^{2}}. \end{array}$$

#### 4.2.2. Nonlinear Method

Considering the effectiveness and stability of this scheme, 5 nonlinear features are extracted from the EEG signal in frequency range (0.5–30 Hz), which includes Shannon entropy, Spectral entropy, Kolmogorov entropy, Max lyapunov exponent, and C0-complexity. In these five EEG features, Shannon entropy ShE(X) is used to describe the amount of information contained in the EEG signal. Spectral entropy SpE(*X*) is used to describe the complexity of the EEG signal. Kolmogorov entropy (KoE) is used to describe the degree of loss rate of the EEG signal. Lyapunov exponent is used to describe the sensitivity to the initial conditions, and Max lyapunov (*λ*_max_) exponent is the most important one of Lyapunov exponents. C0-complexity is used to describe the irregular rate of EEG signal. Among them, Shannon entropy is calculated as follows [38]:

(6) $$\begin{array}{c} \text{ShE}\left(X\right)=-\overset{n}{\underset{i=1}{\sum }}p\left(x\right)\log\;p\left(x\right). \end{array}$$

Spectral entropy is calculated as follows:

(7) $$\begin{array}{c} p\left(i\right)=\frac{P\left(w\right)}{P_{xx,\text{tot}}},\\ \text{SpE}\left(X\right)=-\overset{n}{\underset{i=1}{\sum }}p\left(i\right)\log\;p\left(i\right). \end{array}$$

Kolmogorov entropy is calculated as follows:

(8) $$\begin{array}{c} \text{KoE}=-\underset{\Delta t\to 0}{\lim}\underset{\varepsilon \to 0}{\lim}\underset{n\to \infty }{\lim}\overset{m}{\underset{i=0}{\sum }}p\left(i_{0},\ldots ,i_{n}\right)\log\;p\left(i_{0},\ldots ,i_{n}\right). \end{array}$$

Max lyapunov exponent is calculated as follows:

(9) $$\begin{array}{c} L^{\prime }\left(t_{i}\right)=\min\left|Y\left(t_{i}\right)-Y_{0}\left(t_{i}\right)\right|,\\ \lambda \max=\frac{1}{t_{n}-t_{0}}\overset{n}{\underset{i=1}{\sum }}\log\frac{L^{\prime }\left(t_{i}\right)}{L\left(t_{i-1}\right)}. \end{array}$$

*C*0-complexity is calculated as follows:

(10) $$\begin{array}{c} A_{0}={\overset{n}{\underset{i=0}{\sum }}\left|x\left(i\right)\right|}^{2},\\ A_{1}=\overset{n}{\underset{i=0}{\sum }}{\left|x\left(i\right)-y\left(i\right)\right|}^{2},\\ C0‐\text{complexity}=\frac{{A_{1}}_{}}{A_{0}}. \end{array}$$

The above EEG features are widely acknowledged in the human sleep research. Finally, 50 linear features and 10 nonlinear features are extracted in total.

In order to avoid the effect of EEG features and abnormal values on classification accuracy, each EEG feature is normalized in the range [0, 1] using the Min-Max normalization [39].

(11) $$\begin{array}{c} X_{\mathrm{norm}}=\frac{X-\min}{\max-\min}, \end{array}$$

where *X* represents the initial value of the feature, *X*_norm_ represents the value of the feature after normalization, min represents the minimum value of the feature in a range, and max represents the maximum value of the feature in a range.

### 4.3. EEG-Sleep Ontology

**Figure 2:**
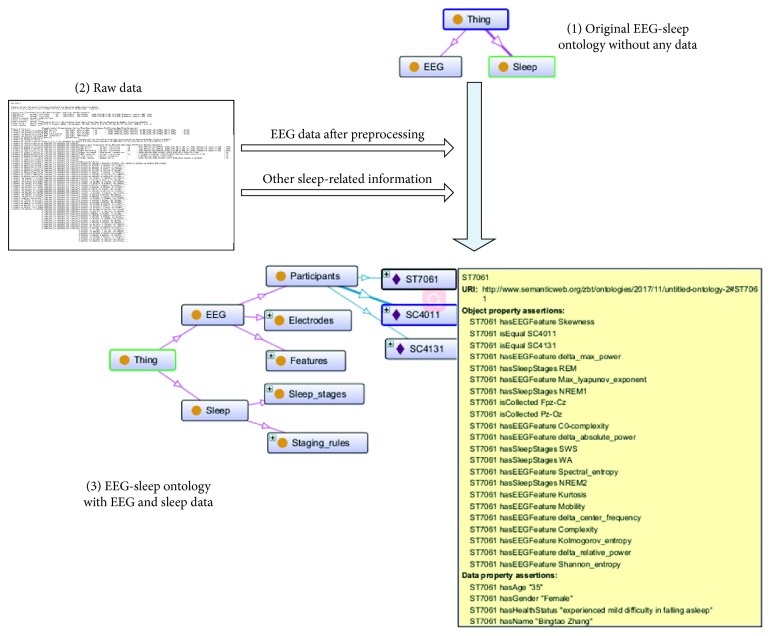
Structure diagram of EEG-sleep ontology.

Once the EEG features are extracted, these features together with sleep-related information are used to form “EEG-Sleep ontology.” This process involves the two aspects of information query mapping and information description. SPARQL language^1^ is used to query the preprocessed EEG features and other sleep-related information from raw data, and then map them to “EEG-Sleep ontology.” In other words, the purpose of the information query mapping is to find the classes and instances required for the original “EEG-Sleep ontology” from raw data. The information description is describing instances in the “EEG-Sleep ontology” using object property and data property. In this study, a top-down approach [40] is adopted to implement information description. A detailed description of this method is shown as Figure 2.

We can see from Figure 2, two categories (EEG and Sleep) are defined in the original “EEG-Sleep ontology,” but the two categories did not contain any instances (Figure 2(1)). Information query mapping is used in both original “EEG-Sleep ontology” and raw data (Figure 2(2)) according to match keyword; the retrieved instance is then mapped to corresponding category in “EEG-Sleep ontology” (Figure 2(3)). Information description provides a clear information representation of “EEG-Sleep ontology.” More specifically, the “EEG-Sleep ontology” is designed as a four-layer structure: domain layer, category layer, class layer, and instance layer. The domain layer is an indispensable component which indicates the name of ontology under modeling. The category layer contains the main categories of OBM. The detail of each category layer is shown in the class layer which contains a set of concepts (class) of OBM. At lowest layer, the instance layer defines all instances (individuals) of each class contained in the class layer. In addition, the relationship among instances is described by object properties. The relationship between instance and the basic data type (i.e., attribute) is described by data properties.

**Figure 3:**
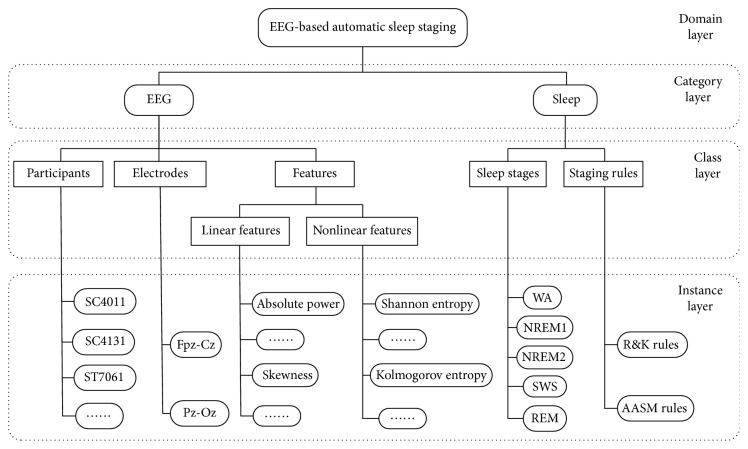
The structure of “EEG-Sleep ontology.”

“EEG-Sleep ontology” can be described as in Figure 3. The domain name: EEG-based automatic sleep staging. This domain defines two categories, named EEG and Sleep, to represent the two main aspects of this study. Next, some concepts related to two categories are defined in the class layer. For example, EEG-related concepts (classes) include participants, electrodes, and features. Sleep-related concepts include sleep staging and staging rules. Finally, each class is materialized to create corresponding instance of the instance layer. As mentioned in the previous paragraph, there are relationships (i.e., object properties) that exist between one instance and another instance. This relationship is used to associate the entire “EEG-Sleep ontology.” For example, an object property “hasEEGfeature” exists between the instance “ST7061” and the instance “Shannon entropy.” Simultaneously, “ST7061” has a data property “hasAge.” The data type of this property is “Integer” and the data value is “35.” Several examples of object properties and data properties for the instance “ST7061” (Figure 2(3)) are listed in Tables 3 and 4.

Next, a correlation analysis is implemented for the instance information stored in the “EEG-Sleep ontology.”

### 4.4. Correlation Analysis

**Figure 4:**
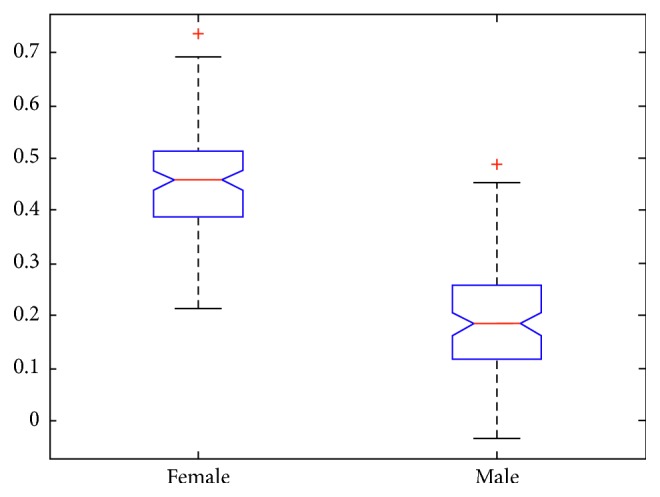
Mean and standard deviation of the Complexity feature for different gender.

An improved algorithm of Pearson correlation coefficient based on self-adaptive correlation analysis is designed (see Algorithm 1 of Section 5.2). Recent studies [41, 42] have found that there are differences in sleep structure between different genders. Therefore, the effect of gender differences on sleep staging is considered. The correlation between EEG features and sleep stages is revealed by gender contrast diagram form. As illustrated in Figure 4, there is a significant difference in the Complexity feature extracted on Fpz-Cz electrode for different gender. It can be see that for female, the Pearson correlation coefficient of Complexity feature is significantly higher than that of male. The results show that the Complexity features have a significant impact on the accuracy of female's sleep staging, but it may have less effect on the accuracy of male's sleep staging.

### 4.5. Classification and Improvement

RF algorithm [43, 44] consists of multiple independent decision trees. Building each decision tree starts at the top of tree with the entire training data. The core task during decision tree-building process is to select a most appropriate attribute (feature) at the node (root node or branch node) and then to split the training data into different subsets based on the attribute.

The criterion of node selection and splitting are done according to an information gain (IG) of the node attribute, and the attribute with the maximum information gain is selected as the splitting criterion. The IG of splitting a training set (*S*) into subset (*S*_*i*_) can be defined as

(12) $$\begin{array}{c} \text{IG}\left(S_{i}\right)=-\underset{i}{\sum }\frac{\left|S_{i}\right|}{\left|S\right|}E\left(S_{i}\right), \end{array}$$

*E*(*S*_*i*_) is the information entropy of the subset *S*_*i*_ calculated as

(13) $$\begin{array}{c} E\left(S_{i}\right)=-\overset{c}{\underset{i=1}{\sum }}p_{i}{\;\log}_{2}\left(p_{i}\right), \end{array}$$

where *c* denotes the number of sleep stages and *p*_*i*_ is a proportion of sleep stage *i* in the subset *S*_*i*_.

Each node attribute is applied to calculate IG, and the attribute with the maximum IG is selected as the splitting criterion. This process is repeated recursively at each node until either every attribute is selected or this process reaches a leaf node that is a classification output.

**Figure 5:**
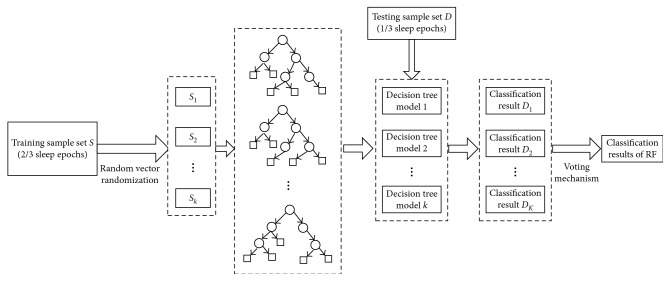
Building RF and classification decision.

Building RF as shown in Figure 5:

1. Each decision tree is generated by a training data *S* with sample size of *k*, and the random vector *θ*_k_ is an independent identical distribution.
2. RF is a set of all decision trees: *h*(*S*, *θ*_k_). Meanwhile, each decision tree is also a model.
3. Each decision tree model *h*(*S*, *θ*_k_) has the right to select a result for the input variable *x*.

(14) $$\begin{array}{c} H\left(x\right)=\underset{Y}{\max}\overset{k}{\underset{i=1}{\sum }}I\left(h_{i}\left(x\right)=Y\right), \end{array}$$

where *H*(*x*) represents the classification result of RF, *h*_*i*_(*x*) is a classification result of a single decision tree, and *Y* is a classification object, that is, the number of sleep stages.

#### 4.5.1. Weighting Feature Analysis and Improved Classification Algorithm

The magnitude of the feature weight reflects the relative importance of each feature in the classification assessment, which indirectly affects the accuracy of automatic sleep staging to a remarkable extent. Traditionally, the calculation methods of feature weight mainly include the Delphi method [45], paired comparison method [46], and so on. However, these traditional methods are generally based on the experience of experts in some fields, and the feature weight coefficient is determined by a prior hypothesis.

The classification process of RF is a partition process based on feature values. It is easy to determine the weight coefficients corresponding to each feature due to the features used for classification are determined before classification. However, except RF and C4.5, it is difficult to determine the corresponding relationship between features and weight coefficients for other classifiers. Additionally, RF consists of multiple decision trees, and its performance is obviously better than C4.5. Therefore, in order to reflect the role of weight coefficients in automatic sleep staging, an improved RF algorithm based on the weight coefficient of standard deviation is proposed. Firstly, we assume that the contribution of each attribute to the classification results is different in a dataset with multiple features. If the standard deviation of an attribute is relatively small, there is little difference in the value of records on the attribute. Meanwhile, the weight coefficient of an attribute is relatively small when the attribute value does not have much effect on the classification results. On the contrary, if the standard deviation of an attribute is relatively large, the weight coefficient of this attribute is also larger. Secondly, on the basis of the first step theory, we calculate the standard deviation after normalization of each attribute in the optimal feature subset according to the following formula:

(15) $$\begin{array}{c} s_{i}=\sqrt{\frac{\sum_{i=1}^{n}{\left(x_{i}-\bar{x}\right)}^{2}}{n}}, \end{array}$$

where $\bar{x}$ represents the mean value of each attribute after normalization.

The weight coefficient of each attribute in the optimal feature subset was calculated according to the following formula:

(16) $$\begin{array}{c} w_{i}=\frac{s_{i}}{\sum_{i=1}^{n}s_{i}}, \end{array}$$

where *s*_*i*_ represents the standard deviation of the *i*th attribute.

Finally, to emphasize the role of the weight coefficient in the classification decision-making process, formula (12) is further improved.

(17) $$\begin{array}{c} {\text{IG}}_{\text{imp}}\left(S_{i}\right)=w_{i}*\text{IG}\left(S_{i}\right)=-\underset{i}{\sum }w_{i}*\left(\frac{\left|S_{i}\right|}{\left|S\right|}E\left(S_{i}\right)\right). \end{array}$$

It is used as a new criterion for the selection and splitting of decision tree nodes in RF after the formula is improved. Selecting new nodes can not only greatly improve the accuracy of the classifier but also reduce the depth of the decision tree. In addition, this improvement can change the outcome of each decision tree, and then it has a positive effect on the classification results of RF.

## 5. Experiment and Discussions

“EEG-Sleep ontology” is used to represent and mange sleep-related information, such as EEG features, participants, and so on. So we first analyze and discuss the relationship between participants' age distribution and sleep staging.

### 5.1. Sample Characteristics of Participants' Age

Means and standard deviations of participants' age are shown in Table 5. Beyond that, Table 5 also shows significant differences of age in different genders. It can be seen that there is no significant difference in the female and male groups for age, *p*=0.16. Thus, we can infer that the effect of age distribution for the sleep-staging accuracy can be neglected in this study.

### 5.2. The Result and Discussion of Correlation Analysis

The sleep-related information and EEG features derived from 61 data records were used to establish “EEG-Sleep ontology.” And then, two sets of optimal feature subsets for different gender were calculated from EEG features of 1/2 which were stored in “EEG-Sleep ontology” using improved Pearson correlation coefficient. That is, half of sleep segments for female (14753 sleep segment) and male (10867 sleep segment) were used to calculate optimal feature subsets.

**Figure 6:**
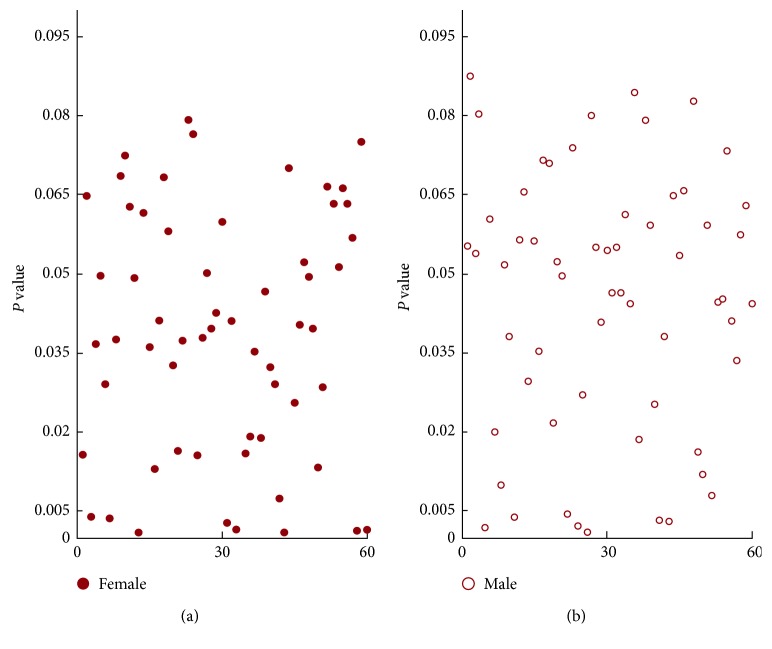
Scatter plot of correlation analysis results for 60 EEG features.

The results of Pearson analysis for 60 EEG features are shown in Figure 6. In this experiment, we selected the threshold of *p*=0.005, and the calculation process of self-adaptive threshold value *p* are shown in Algorithm 1. The salient channels and features were obtained using improved Pearson analysis, and the result is reported in Table 6. As you can see in Table 6, the optimal feature subset of female contains 8 features, while the optimal feature subset of male contains 7 features. The most relevant features of both male and female were mainly derived from EEG linear features, and this result indirectly shows that linear features were useful for understanding the potential sleep structure.

### 5.3. Classification and Comparison Analysis

To find an effective classification algorithm, we have designed the following experiment: (1) We adopt optimal subset of different gender in Table 6 as the input of five classical classification algorithms to classify five-state sleep stages (WA, NREM1, NREM2, SWS, and REM). (2) Seven performance evaluation indexes were calculated for different genders, respectively. (3) Different classification algorithms were compared, the purpose of which was to observe the accuracy of the classification results obtained by the different classifiers in different evaluation indexes.

Five classical classification algorithms, with the exception RF, also include Bayesian network (BN) based on the graph model of probability, decision tree (C4.5) based on information entropy theory, multilayer perception (MLP) based on logistic regression, and support vector machine (SVM) based on statistical learning theory. Seven indicators of performance evaluation [47] involved in this experiment include the following: (1) Accuracy (AC) is defined as TP + TN/(TP + TN + FP + FN) (2) The true positive (TPR) or sensitivity is defined as TP/(TP + FN); it is also called the recall in some fields. (3) The true negative rate (TNR) or specificity is defined as TN/(TN + FP). (4) Precision defined is defined as TP/(TP+FP). (5) F-measure is defined as 2 ∗ Precision ∗ Recall/(Precision + Recall). (6) ROC Area is a combination of sensitivity and specificity under different threshold. (7) Kappa statistic is defined as 2 ∗ (TN ∗ TP-FP ∗ FN)/((TN + FN) ∗ (FN + TP) + (FP + TP) ∗ (TN + FP)).

Weka software package [48] was used to perform all the classification tasks in the experiment. To save the running time of RF, we modified the number of decision tree from 100 to 30. The remaining parameters of RF and the parameters of other classifiers use the default values. The detailed usage of data in this experiment is shown in Table 7.

**Figure 7:**
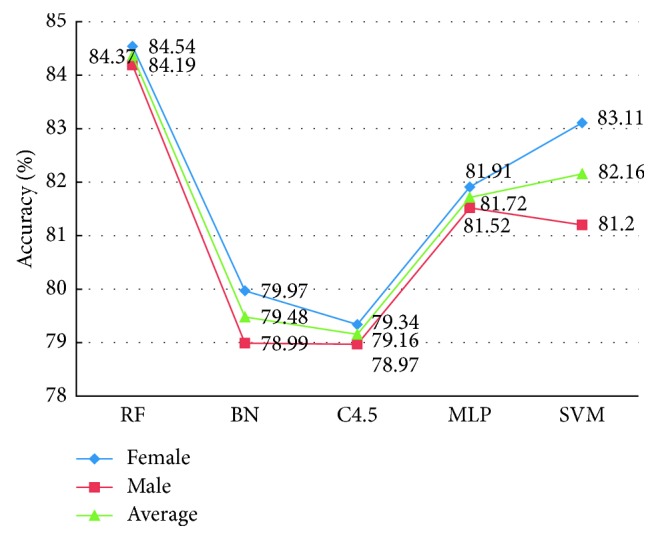
The classification accuracy of different classifiers on five-state sleep stages.

A standard experiment was implemented 10 times in Weka using the above scheme, and Figure 7 illustrates the average of classification accuracy of different classifiers for five-state sleep stages. For the classification accuracy, there were some differences between the genders, but this difference has little effect on the results of sleep staging. So in the follow-up experiments, we only considered the average accuracy and ignored gender differences.

From Figure 7, it appears that the best average classification results from the RF with an accuracy of 84.37%. Why the RF classifier is superior to other classifiers? We believe that the main reason can be attributed to as follows:

(1) The randomness of RF makes the structure of each decision tree not exactly the same, and the comprehensive voting will greatly improve the classification accuracy. For example, suppose that an RF was made up of three decision trees, the error rate of each tree was 40%, and the error rate was reduced to 35.2% after comprehensive voting. Therefore, the accuracy of RF classification was obviously higher than that of C4.5.
  (18) $$\begin{array}{c} 1\times 0.4^{3}+3\times 0.4^{2}\times {\left(1-0.4\right)}^{1}=0.352. \end{array}$$
(2) Ensure that the data in each record have an equal use probability. The data for this study were not continuous and just a random integration of data from all night (Section 2). The data use strategy was consistent with the random sampling mechanism of RF [18], which enables RF to have strong generalization ability, and it can dig out the hidden rules behind the data, so as to obtain a high classification rate.

**Figure 8:**
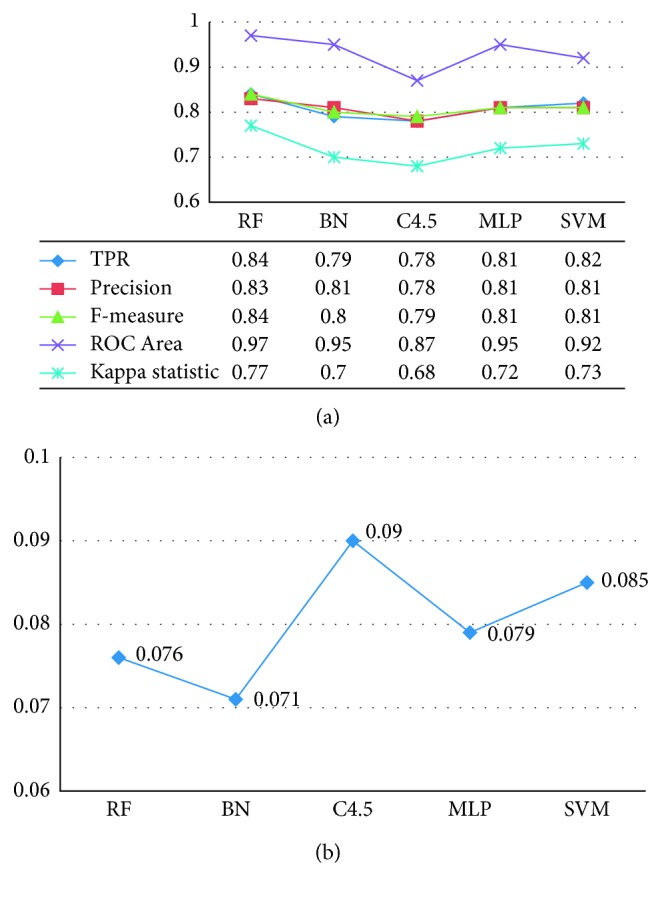
The experimental comparison results of six performance indexes on five classifiers. (a) TPR, Precision, F-measure, ROC Area, and Kappa statistic. (b) TNR.

To confirm this result, we also compared and analyzed the remaining six performance evaluation indexes, and the specific experimental results are shown in Figure 8. For these six indexes, we expect higher values of TPR, Precision, Recall, F-measure, and Kappa statistic and lower value of TNR because these mean a better classification result. As we expected, the experimental results support our prediction that RF can achieve higher TPR, Precision, F-measure, ROC Area, and Kappa statistic than other four classification classifiers. However, it is remarkable that TNR of BN is a little lower than RF. But based on a comprehensive comparison of the various aspects (including accuracy and other five evaluation indicators), we finally selected the RF as classifier to identify different sleep stages.

### 5.4. Results of Improved Classification Algorithm

The core idea of weighting feature analysis and algorithm improvement has been previously explained in Section 4.5.1. The following was a specific process of experimental design and implementation: (1) We eliminated the 25620 sleep segments used in Section 5.2 correlation analysis and selected the remaining half of sleep segments as a sample library of weighting feature analysis and classification. This was done to prevent overfitting with the above experiments of correlation analysis. (2) The weight coefficient obtained in the first step was used as the input of formula (17) and applied to the calculation of the IG in the subsequent experiments. (3) The results of sleep staging in different cases were tested.

Based on formulas (15) and (16), the standard deviation and weight coefficient of each feature in Table 6 were calculated, and the results are shown in Tables 8 and 9.

To investigate the performance of the proposed method, the following five cases were tested. Meanwhile, to ensure the scientificity and rationality of the follow-up experimental comparison, we applied the other half of the data except Section 5.2. This is done to keep that in consistence with data used to calculate the weight coefficient, and the detailed data usage of the experiment is shown in Table 10. The Matlab2Weka toolbox was used to implement RF classification tasks. In addition to the improved part of algorithm, the number of decision trees in RF is still 30, and the other parameters were set as the default values.

*Case 1*. Five-state sleep stages were classified based on RF without weight coefficient.

*Case 2*. Five-state sleep stages were classified based on RF with weight coefficient.

*Case 3*. Four-state sleep stages were classified based on RF with weight coefficient.

*Case 4*. Three-state sleep stages were classified based on RF with weight coefficient.

*Case 5*. Two-state sleep stages were classified based on RF with weight coefficient.

We only list the average performance evaluation results of sleep staging in these five cases. This was done to facilitate the comparison of the subsequent experimental results. And beyond that, the experiment of Section 5.3 also showed that there was little difference in the results of sleep staging between different genders. Based on the above five cases, the five sets of standard experiment was conducted 10 times, respectively, using the data from Table 10.

The confusion matrix and average sensitivities of the first two cases are listed in Tables 11 and 12. In these two tables, each column represents sleep stage as predicted by classifier, while each row represents the actual classification of sleep stage by well-trained technicians according to the R&K manual. From the experimental results of the both tables, it is obvious to observe that the classification sensitivity is improved after adding the weight coefficients to RF. In particular, the sensitivity of REM stage was improved most obviously (79.00% up to 88.27%). The most noticeable results in these two tables were that the lowest classification sensitivity was related to the NREM1 stage, with 58.98% and 63.21%, respectively. Considering the result of NREM1, while it represents the lowest classification sensitivity, this result may be expected because of the following: (1) NREM1 and REM exhibit similar EEG wave patterns. (2) NREM1 was a transition phase of WA and NREM2 [49]. The result was consistent with the conclusion reported in literatures [50, 51] that NREM1 stage was easily mistakenly categorized as any of WA, NREM2, and REM stages. More importantly, the results of Tables 11 and 12 verify this conclusion: NREM1 stage was mainly mistakenly classified into WA, NREM2, and REM stages.

Table 13 presents the classification accuracies and kappa statistics for the five cases. The average accuracy was 84.31% in Case 1 and kappa *k* was 0.77, while the average accuracy was 88.98% in Case 2 and kappa *k* was 0.82. From comparison results of Case 2 to Case 5, it is clear that the classification accuracy increases with the decrease of sleep categories. And the classification accuracies were 88.98%, 89.73%, 92.78%, and 95.57%, respectively.

### 5.5. Classification Accuracy and the Number of Sleep Segments

Case 1 in Table 13 compared with Figure 7, we can observe that the accuracy of sleep staging has changed with the decrease in the number of sleep segments. To further analyze this result, the following experiment was conducted to test the accuracy of sleep staging under different numbers of sleep segments. The selected segments (25620, 32025, 38430, 44835, and 51240) were divided into two sets, the training (2/3) and testing (1/3) sets. Then, the weight coefficients were calculated and used as input of RF (with weight coefficient) to classify five-state sleep stages.

**Figure 9:**
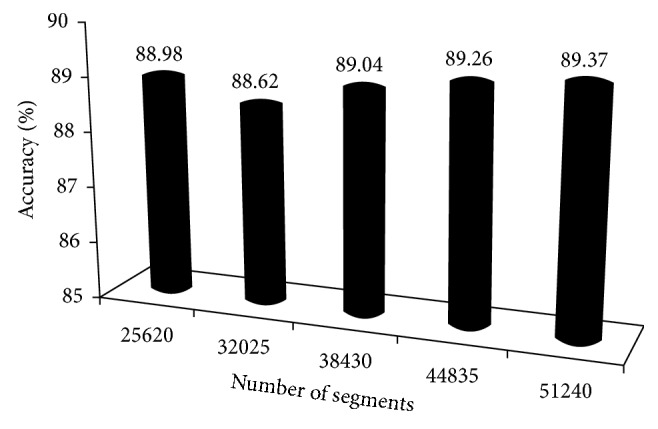
Accuracy comparison based on the number of sleep segments.

For the classification accuracy, Figure 9 shows a comparison of classification accuracy among different numbers of sleep segments when the improved classification algorithm was adopted. According to the obtained results, there are no significant differences in the average classification accuracy among 5 different number of sleep segments. The classification accuracy was ranged between 88.5% and 89.5%. So it was indirectly revealed that the improvement classification algorithm of our proposed method was less affected by the number of sleep segments. Weighting feature analysis used in RF achieved the highest classification accuracy. It improves the classification accuracy by 5.00% (from 84.37% to 89.37%). If the correlation analysis was not considered, the raising space of accuracy rate should be higher.

### 5.6. Comparison with Existing Methods

It is difficult to compare the effectiveness of various methods for automatic sleep staging because of the differences among datasets and the differences in the specific use of data. To mitigate the impact of these problems on performance comparison, our proposed method was tested on the Sleep-EDF database [34, 52] which is a subset of the Sleep-EDF database (EXPANDED) and widely used in many studies. This dataset contains 8 data recordings: sc4002e0, sc4012e0, sc4102e0, sc4112e0, st7022j0, st7052j0, st7121j0, and st7132j0. The performance of the proposed method is compared to that of the previously published studies.  Table 14 provides performance comparison of various state-of-the-art methods that utilize Sleep-EDF dataset.

Based on our proposed scheme, the comparison between Tables 13 and 14 can be observed that classification accuracy from Sleep-EDF database yields better than Sleep-EDF database (EXPANDED). We think that this result may be caused by data quality. However, this conclusion is the only conjecture, and it needs further verification by sleep experts. As shown in Table 14, for four-state sleep stages and three-state sleep stages, our proposed method outperforms others cases. For five-state sleep stages and two-state sleep stages, it gains high accuracy despite not surpassing the accuracy of some prior studies.

### 5.7. Discussion

In this section, we first discuss the main advantages of the proposed scheme, which makes it superior to other existing automatic sleep staging methods.

1. Multimodal physiological signals fusion-based methods (EEG, EOG, ECG, EMG, etc.) [24, 53, 54] suffer from some limitations. For example, EOG and ECG recordings require sticky electrode placement, and this often poses restrictions on the subject's movements [6, 13]. Meanwhile, positions of subjects frequently interfere with the wire, thus diminishing the quality of EOG, ECG, and EMG signals. To overcome these limitations, we have proposed the EEG-based method.
2. A major roadblock in sleep staging based on visual inspection is the identification of the sleep stages NREM1 and REM. In fact, it is also a notable disadvantage of some existing automatic sleep-staging schemes. As in [9, 13], a sensitivity of 18.75% and 15.80%, respectively, are reported for NREM1 in five-state sleep staging. Aiming at this problem, a salient feature of our proposed method is that it can effectively identify REM. As seen from the confusion matrix in Table 12, the proposed method gives a sensitivity value of 63.21%, which is much higher than those of [9, 13].
3. “EEG-Sleep ontology” is designed and applied to manage a huge bulk of sleep EEG features and other context information. Its major advantage lies in the realization of massive information's hierarchical management, which is convenient for human's reading and computer inquiries.
4. A large number of EEG features (more than 50) have been used in studies [55] to achieve high classification performance. In this study, an improved method of self-adaptive correlation analysis is proposed to select the most effective EEG features. This method significantly reduces the number of selected features. For each subject, the number of selected features is less than 10.
5. Weighting feature analysis-based improved RF not only greatly improves the classification accuracy but also avoids the overfitting in a certain extent because each tree of the forest only selects partial features.

From the above discussions, we can know that our work has made some achievements. Additionally, there are also some disadvantages. For example, under the same conditions, the dual-channel EEG sleep-staging method adopted in this paper increases the computational cost compared to the single-channel EEG. To solve this problem, on the one hand, we can use the advanced feature selection algorithm (such as an improved method of adaptive correlation analysis proposed in this study) to reduce feature dimension and computational cost. On the other hand, with the rapid development of hardware technology, we can apply advanced hardware acceleration technology to make up for the dual-channel computing cost problem. For example, the proposed algorithm can be implemented in Compute Unified Device Architecture- (CUDA-) based Graphical Processing Unit (GPU) to speed up the whole process. Although the dual-channel EEG increases the computational cost, we can effectively solve it by the above two methods. We thus conclude that our proposed scheme for automatic sleep scoring is effective and efficient. In addition, we need to explain that the single-channel ideas adopted in document [9, 10, 13] are worthy of our learning and reference. In the future, sleep stages based on single-channel EEG will become one of our research directions.

## 6. Conclusion and Future Work

In this paper, an automatic sleep-staging method based on EEG is proposed. The ontology-based model and the weighting feature analysis are used to represent related information of sleep staging and to improve classification algorithm, respectively. It can be seen from this paper that OBM can use a few simple terms to express and manage most of the sleep-related information, so as to solve the problem of information structured management in the process of automatic sleep staging. An improved correlation analysis algorithm based on self-adaptive correlation analysis is designed to explore the optimal subsets of different genders. And the experimental results show that linear features were useful for understanding the potential sleep structure. More importantly, the improved RF algorithm based on weighting feature analysis increases the classification accuracy by 5%. We not only verified the validity of this method by experiments but also analyzed and summarized the effects of the number of sleep segments on the classification results. Furthermore, our proposed scheme yields good NREM1 detection accuracy.

As we have shown, in the future work, our proposal scheme is feasible and potentially usable in real world for clinical assistant diagnosis of sleep disorders. Meanwhile, our classification method can be extended to other classification problems based on physiological signals, such as depressive, OSA, epilepsy, and so on. Additionally, we shall focus on exploring deep learning techniques to further improve the classification performance. To conclude, we can anticipate that our proposed method of automatic sleep staging will alleviate the burden of sleep technicians and benefit humans.

## Figures and Tables

**Algorithm 1:**
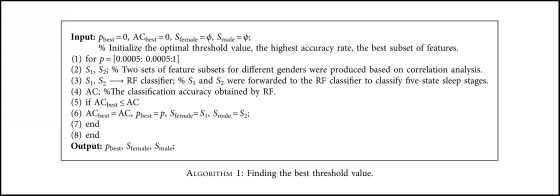
Finding the best threshold value.

**Table 1:** Different combination criterions of six sleep stages.

Combination criterion	Sleep stages
Six-state	WA, NREM1, NREM2, NREM3, NREM4, REM
Five-state	WA, NREM1, NREM2, SWS, REM
Four-state	WA, light sleep, SWS, REM
Three-state	WA, NREM, REM
Two-state	WA, sleep

*Note*. Light sleep: NREM1 and NREM2 are pooled into a single stage. SWS: NREM3 and NREM4, are pooled into a single stage. Sleep: NREM1, NREM2, NREM3, NREM4 and REM are pooled into a single stage.

**Table 2:** Specific database usage in this study (unit: epoch or segment).

		WA	NREM1	NREM2	SWS	REM	Total
SC∗PSG.edf	Females	1397	1476	7557	3864	2540	16834
	Males	1292	919	7675	3529	2523	15938

ST∗PSG.edf	Females	924	1163	5774	2925	1886	12672
	Males	529	554	2809	1282	622	5796
	Total	4142	4112	23815	11600	7571	51240

**Table 3:** Several objective properties of the instance “ST7061.”

Instance 1	Objective properties	Instance 2
ST7061	hasEEGfeature	Shannon entropy
ST7061	hasSleepStages	REM
ST7061	isCollected	Pz-Oz

**Table 4:** Several data properties of the instance “ST7061.”

Instance	Data properties	Value	Data type
ST7061	hasAge	35	Integer
ST7061	hasGender	Female	String
ST7061	hasHealthStatus	Mild difficulty in falling asleep	String

**Table 5:** Basic information on the age of participants.

	Mean	SD	*p* value
Female	34.74	13.78	*p*=0.16
Male	30.27	6.04	

*p* < 0.01: extremely significant difference. 0.01 < *p* < 0.05: significant difference. *p* > 0.05: no difference.

**Table 6:** The significant EEG features of different gender based on Pearson correlation (*p* < 0.005).

Gender	Electrode	Features
Female	Fpz-Cz	Delta relative power, Complexity, Activity, Sawtooth relative power
	Pz-Oz	Delta relative power, Activity, Mobility, Skewness

Male	Fpz-Cz	Delta relative power, Alpha center frequency, Complexity, Skewness
	Pz-Oz	Delta relative power, Alpha center frequency, Kurtosis

**Table 7:** Data usage in this experiment (unit: epoch or segment).

	Training data	Testing data	Total
Female	19671	9835	29506
Male	14489	7245	21734
			51240

**Table 8:** Standard deviation and weight coefficient of different features (female).

Feature	*P*_rel_ (*C*z, *δ*)	Com (*C*z)	Act (*C*z)	P_rel_ (*C*z, saw)	P_rel_ (*O*z, *δ*)	Act (*O*z)	Mob (*O*z)	Skew (*O*z)
Standard deviation	0.373142	0.198726	0.265727	0.305066	0.171219	0.352091	0.252303	0.144717
Weight coefficient	0.180874	0.096328	0.128807	0.147876	0.082996	0.170670	0.122300	0.070149

*Note*: *P*_rel_ (Cz, δ): the relative spectral power of delta on Fpz-Cz electrode; Com (*C*_z_): complexity on Fpz-Cz electrode; Act (*C*_z_): activity on Fpz-C_z_ electrode; *P*_rel_ (*C*_z_, saw): the relative spectral power of sawtooth on Fpz-Cz electrode; *P*_rel_ (*O*_z_, δ): the relative spectral power of delta on Pz-Oz electrode; Act (*O*_z_): activity on Pz-Oz electrode; Mob (*O*_z_): mobility on Pz-Oz electrode; Skew (*O*_z_): skewness on Pz-Oz electrode.

**Table 9:** Standard deviation and weight coefficient of different features (male).

Feature	*P*_rel_ (*C*_z_, *δ*)	*P*_cent_ (*C*_z_, *α*)	Com (*C*_z_)	Skew (*C*_z_)	*P*_rel_ (*O*_z_, *δ*)	*P*_cent_ (*O*_z_, *α*)	Kurt (*O*_z_)
Standard deviation	0.378916	0.335212	0.254561	0.183792	0.381092	0.314814	0.242165
Weight coefficient	0.181252	0.160346	0.121767	0.087915	0.182293	0.150589	0.115838

**Table 10:** Data usage in this experiment (unit: epoch or segment).

	Training data	Testing data	Total
Female	9835	4918	14753
Male	7245	3622	10867
			25620

**Table 11:** Confusion matrix and average sensitivity on five-state sleep stages (without weight coefficient).

		Classifier					
		WA	NREM1	NREM2	SWS	REM	Sensitivity (%)
Experts	WA	581	51	37	7	15	84.08
	NREM1	64	404	123	5	89	58.98
	NREM2	71	137	3459	184	118	87.15
	SWS	5	3	164	1759	2	91.00
	REM	24	74	154	13	997	79.00

**Table 12:** Confusion matrix and average sensitivity on five-state sleep stages (with weight coefficient).

		Classifier					
		WA	NREM1	NREM2	SWS	REM	Sensitivity (%)
Experts	WA	594	46	33	6	12	85.96
	NREM1	59	433	109	3	81	63.21
	NREM2	64	99	3645	105	56	91.84
	SWS	4	3	111	1813	2	93.79
	REM	6	31	97	14	1114	88.27

**Table 13:** Classification accuracies and Kappa statistic based on the five cases.

Case	Case 1	Case 2	Case 3	Case 4	Case 5
Accuracy rate (%)	84.31	88.98	89.73	92.78	95.57
Kappa statistic	0.77	0.82	0.82	0.85	0.89

**Table 14:** Performance comparison of various state-of-the-art methods in terms of accuracy (%).

Researchers	Five-state (%)	Four-state (%)	Three-state (%)	Two-state (%)
Sharma et al. [6]	**91.70**	92.10	93.90	**98.30**
Liang et al. [9]	83.60	—	—	—
Sharma et al. [10]	91.13	92.29	94.66	98.02
Zhu et al. [13]	88.90	89.30	92.60	97.90
Hsu et al. [17]	87.20	—	—	—
Berthomier et al. [23]	71.20	74.50	88.30	95.40
Our proposed method	90.57	**92.41**	**94.85**	97.21

Boldface indicates that the highest classification accuracy values. “—” indicates the missing cases.

## Data Availability

The data used to support the findings of this study are available from the corresponding author upon request.
